# Development and Content Validation of The Health Promoting Sports Club Observational Tool

**DOI:** 10.34172/hpp.45562

**Published:** 2026-06-06

**Authors:** Stacey Johnson, Petra Ozbolt, Louana Friche, Kevin Barros, Florance Rostan, Thierry Fauchard, Aurélie Van Hoye, Susanna Geidne, Anne Vuillemin

**Affiliations:** ^1^Laboratory UMR1319 INSPIIRE, Faculty of Sport Sciences, Université de Lorraine, Vandœuvre-Lès-Nancy, France; ^2^Laboratory LAMHESS, Faculty of Sports Sciences, Université Côte d’Azur, Nice, France; ^3^Department of Prevention and Health Promotion, Santé Publique France, Saint-Maurice, France; ^4^Sports, Health and Wellness Resource Centre, French Ministry of Sports, Youth and Community Life, Vichy, France; ^5^Department of Physical Education and Sport Sciences, PAH Research Center, University of Limerick, Limerick, Ireland; ^6^Faculty of Medicine and Health, School of Health Sciences, Örebro University, Örebro, Sweden

**Keywords:** Delphi study, Health promotion, Observational tool, Rapid review, Sports clubs

## Abstract

**Introduction:**

A lack of rigorous, objective measurement of health promotion (HP) in sports clubs has been identified. This study developed and validated content of an observational tool for sports managers to evaluate HP within their clubs.

**Methods:**

First, a rapid literature review identified sports club HP observational tools and their development process. Second, a 3-round Delphi study gathered feedback from 69 sports stakeholders to design a checklist. Round 1 created items and observation areas; Round 2 obtained feedback, item suggestions and reformulations; Round 3 structured items into a checklist and rated them on three indicators. Third, experts categorized items into health determinants from the health promoting sports club (HPSC) model and drafted a user’s guide.

**Results:**

The rapid review yielded 11 studies, including tools for injury surveillance, coaching behaviours, alcohol management, referee-athlete interaction and motivation, but none directed at HP. The Delphi study round 1 included 18 respondents formulating 24 items organized into: Locker room, Website, Cafeteria & Food Offerings and Facilities & Surroundings. Round 2 included 31 respondents validating 43 items and reformulating ‘Locker room’ into ‘Locker and Restrooms’. Round 3 included 21 respondents validating 36 items separated into the four areas. Five researchers categorized items into Economic (n=5), Environmental (n=16), Organizational (n=17) and Social (n=9) health determinants. The HPSC Observational Tool includes a user’s guide, a checklist including scoring methods and recommendations.

**Conclusion:**

This study provides an innovative observational tool for sports managers to assess their club’s HP. Sports managers can directly evaluate specific areas and follow recommendations for improvement.

## Introduction

 Sports clubs welcome a large population of European citizens providing an informal educational environment making them an ideal setting for a range of health benefits.^1,2,3^ Sports clubs traditionally provide “opportunities for competition and sports practice, while some can also be considered social organisations, promoting social welfare and health”.^2^ A 2025 literature review reported that health promotion (HP) interventions in sports clubs may increase physical activity and healthy dietary intake.^4^ Nevertheless, research also demonstrates that sports clubs can be unhealthy settings^5^ when considering commercial health determinants^6^, mental health issues,^7^ injury risk,^8^ or contributing to eating disorders.^9^ Therefore, considering sports practice as automatically healthy^10^ is reductive and efforts to improve the actual setting that delivers sports including the social and built environment should be developed and implemented.

 Sports clubs can potentially^11^ become comprehensive health-promoting settings, defined as “*the place or social context where people engage in daily activities in which environmental, organizational and personal factors interact to affect health and well-being*”.^12^ The settings-based approach applied to sports clubs, called Health Promoting Sports Clubs (HPSC), moves beyond single health topic interventions (i.e., physical activity, healthy eating, etc.), towards a socio-ecological approach by embedding health into daily club actions.^13^ Similar to other settings for HP, this approach encourages sports clubs to target health determinants at multiple levels when planning and implementing HP interventions.^14,15^ The theoretical HPSC model includes a dedicated level for sports club managers, being volunteers or employees responsible for organising and structuring sport practices within the setting.^13,16^ HPSC determinants include economic (financial, human, material resources), environmental (built surroundings), organizational (guidelines, regulations, actions) and social (vision, values, ideologies), each dynamically interacting at all levels to promote health throughout the sports club and wider community.^17^

 Currently, research on HPSC relies on cross-sectional, self-reported studies with the settings-based approach being poorly implemented.^18^ One review identified a lack of validated measurement instruments to evaluate HP in sports settings,^19^ as well as low quality study design^4^ and insufficient use of theory-based implementation methods to evaluate interventions.^20^ Existing evaluation tools, such as the Health Promotion Assessment Tool in sport (HP-SAT)^21^ or the e-PROSCeSS questionnaires^22^ are self-reported raising concern about validity and reliability^23^ in sports contexts. Self-report instruments are prone to recall and social desirability biases and inaccuracies.^23^ These limitations have been widely acknowledged in the settings-based HP literature, which also highlights inconsistencies in how HP practices are measured across countries and contexts.^24^ Moreover, while validating the e-PROSCeSS questionnaire, the environmental determinant was poorly understood raising concerns about how this health determinant can be mobilized in sports clubs.^22^ Thus, there remains a lack of a well-designed tool to assess the presence and extent of HP within sports club. Observational tools capture real-world behaviours and contextual nuances often overlooked by self-report instruments and can thus be critical to assessing contextual HP. Additionally, observational tools record observations which can infer reality from an external point-of-view without requiring verbal or written explanations. These indications can be systematically observed and compared over time and made more complex by layering information.^25^ For example, they have been used to observe neighbourhood walkability and the food and physical activity environment in schools.^26,27^ Given the usefulness of direct observational tools and the unrealized potential of sports clubs to fully embrace the HPSC approach, there is a need to develop a robust, internationally applicable observational tool designed to evaluate how sports clubs promote health. An observational tool based on the HPSC model that incorporates specific levels and health determinants, notably the environmental determinant, will provide sports club actors with a tangible method to directly observe and record HP. This allows for sports club actors to visualize and record potential areas to improve their health-promoting potential. Furthermore, sports club actors have requested support including practical guidance and tools when implementing and evaluating HP within their club.^28-30^ To fill this need, the present study aimed to develop and validate content of a theory-based HPSC observational tool designed for sports management to directly observe, record and improve HP in their sports clubs.

## Methods

###  Design

 To determine and employ best-practice in HP observational tool development, a 3-step process was undertaken: 1) a rapid literature review to examine existing observational tools evaluating HP in sports settings and their development process, 2) a modified Delphi study to gather expert and sports manager opinions regarding areas to observe within a sport club and items to include on a checklist and 3) expert categorization of items into HPSC health determinants,^13^ development of an evaluation system and drafting a user’s guide.

####  Step 1: Existing Observational Tool Rapid Review

 The first step gathered information on observational tools that measure HP in sports settings to understand what was measured, tool development, validation and implementation methods. A rapid review was preferred as the objective was to map existing observational tools and their development process rather than systematically investigating their effectiveness and associated results.^31,32^ This method allowed for suffiecient knowledge gained and used for purposes of this study while maintaining the timeline and ensuring rigor.^33^

#####  Search Strategy

 The search for relevant studies was conducted in PubMed, SPORTDiscus, Scopus and Web of Science between December 2024 to February 2025. Studies were considered for inclusion if they 1- were peer-reviewed articles published in English after 1986,^34^ 2- focused on HP within organized sports settings and 3- developed and tested an instrument to systematically record and assess observable behaviours, actions or conditions in sports clubs. Studies were excluded if they focused on non-health-related variables, were from grey literature or focused on elite level sports or sports performance metrics.

 Search terms combined the following key words: (1) “observational tool”, “assessment tool”, “evaluation tool”, (2) “health”, “health promotion”, “health behaviour”, “well-being”, and (3) “sports settings”, “sports clubs”, “community sports”.

#####  Data Analysis

 Article selection was completed by 2 researchers (LF, KB) using Rayyan with a third author (AVH) arbitrating disagreements. A narrative synthesis approach was used for article analysis to identify and extract key characteristics.^35^ The following information was extracted and added to an Excel spreadsheet: authors, date, country, population targeted, tool description, validation method, tool content, targeted health determinant, theme, HPSC level targeted and quality rating. The quality assessment used a 4-point rating system with 1 indicating ‘poor’, 2 indicating ‘fair’, 3 indicating ‘good’ and 4 indicating ‘excellent’. Risk of bias (low, moderate, high) was determined based on methodological quality. Key assessment criteria for study quality and risk of bias included population representativeness, data collection consistency and validity, clarity and relevance of outcome measurements, statistical transparency and identification of bias and confounding factors. This approach prioritized studies with high methodological quality and minimized the risk of bias with potential methodological weaknesses consistent with the Joanna Briggs Institute (JBI) critical appraisal tool. Data extraction and ratings followed JBI methodologies.^36^ The targeted HPSC levels and health determinants were guided by the HPSC approach,^13,16^ while tool themes were categorized using inductive thematic analysis.^37^

####  Step 2: Delphi Study

 To develop and validate content on a checklist for the observational tool, based on results from the literature review, the HPSC approach^13,16^ was selected as a framework for its relevance and robustness. This approach encourages promoting multiple health topics encompassing four health determinants at multiple sports club levels.^2,16^ Furthermore, it considers HP as a process,^17,38^ was developed from a Delphi study^39^ and used as the basis for the e-PROSCeSS questionnaire.^22,39^ Due to low evidence on the effectiveness of HP interventions in sports contexts^40^ and high importance of having systematic consensus to gather quality data, a modified Delphi study^41^ was chosen to elicit expert and sports manager opinions on items and areas to include on a structured observational checklist.

#####  Participants

 Experts from the ‘Promoting physical activity and health in sports clubs’ Health Enhancing Physical Activity (HEPA) Europe working group were invited to participate in all rounds. Experts have an academic, sport or public health profile, are located across Europe, have at least 5-years of experience in research on HP in sport and a working knowledge of English. Additionally, to gather field actor insights, sports managers were invited through the author’s networks and participating HEPA expert networks to rounds 2 and 3 ensuring end-user input and usability. Prior to each round, respondents completed an informed consent. Participation was anonymous and respondents could withdraw at any stage.

#####  Procedure

 The Delphi method structures group communication, allowing for an effective process of reaching group consensus to solve complex problems.^42^ This method was previously used in sports club contexts for questionnaire^39^ and health policy^43^ development. It establishes rounds of questions with each round building upon previous responses.^42^ For this study, three rounds were conducted including item generation, selection, reformulation, ratings and validation. During a preparation meeting, the research team formulated an initial item checklist separated into observable sports club areas framed by the HPSC approach,^13^ using elements from the rapid review, the e-PROSCeSS questionnaire^22^ and the HP-SAT.^21^

 Round 1 was conducted during an online meeting in February 2025, where 27 HEPA experts were invited to give feedback on proposed items, generate additional items based on HPSC health determinants and decide upon specific areas within sports clubs to observe and determine rating indicators. Three rating indicators were defined for subsequent rounds: HP relevance (*How relevant is the item regarding health promotion in sports clubs?*), feasibility (*How feasible/doable is this item for sports clubs?*) and importance (*How important is this item regarding other priorities for sports clubs?*). Due to participant’s countries of origin, items were generated in English and then forward and back translated into French by native French speakers from the research team.

 Rounds 2 and 3 were conducted online in April and May 2025, respectively, inviting the 27 HEPA experts and 42 sports managers to respond to a web-based Limesurvey^TM^. Respondents were given 3 weeks to respond with a reminder email sent 1-week prior to each round closing. Surveys were provided in English and French with respondents encouraged to use translation software for other native languages. Respondents were invited to add and reformulate items, offer opinions on areas to observe, give general feedback or suggest changes to the checklist layout and rate items based on indicators from round 1. Ratings were based on a 6-point Likert scale (1 = Do not agree at all to 6 = Totally agree).

#####  Data Analysis

 An Excel spreadsheet was used to calculate item median scores and interquartile ranges (IQR). For rounds 2 and 3, descriptive statistics were applied to assess consensus; items were retained at ≥ 80% consensus for all three indicators. Strong consensus was described as responses receiving a mean score of ≥ 4 on the Likert scale and an IQR ≤ 1, moderate consensus to any mean score ≥ 3.99, or an IQR ≤ 1.25 (26). Items < 80% for two indicators were deleted and items < 80% for one indicator underwent qualitative analysis with three researchers agreeing to keep, reformulate or exclude items. Consistency of ratings between rounds was investigated, with a cutoff set at a 10% deviation.^44^

####  Step 3: Item Categorization and User Guide Development

 Based on the HPSC model and framework,^13^ study authors were invited to individually categorize retained items into one or more of the HPSC health determinants. Categorization was based on HPSC health determinant definitions and evidence-based strategies used to employ determinants at the sports club and management level of the HPSC theoretical model,^13^ the HPSC logic model^17^ and author’s previous experience in the field of HPSC research. Once individual categorizations were completed, a finalization meeting was conducted with all authors where discrepancies were discussed and consensus reached if two or more authors categorized the item into the same health determinant. Where relevant, items were categorized into multiple health determinants. A final HEPA expert meeting drafted a user’s guide, developed an evaluation system and formulated evidence-driven recommendations for improvement.

## Results

###  Step 1: Rapid Literature Review Results

**Figure 1:**
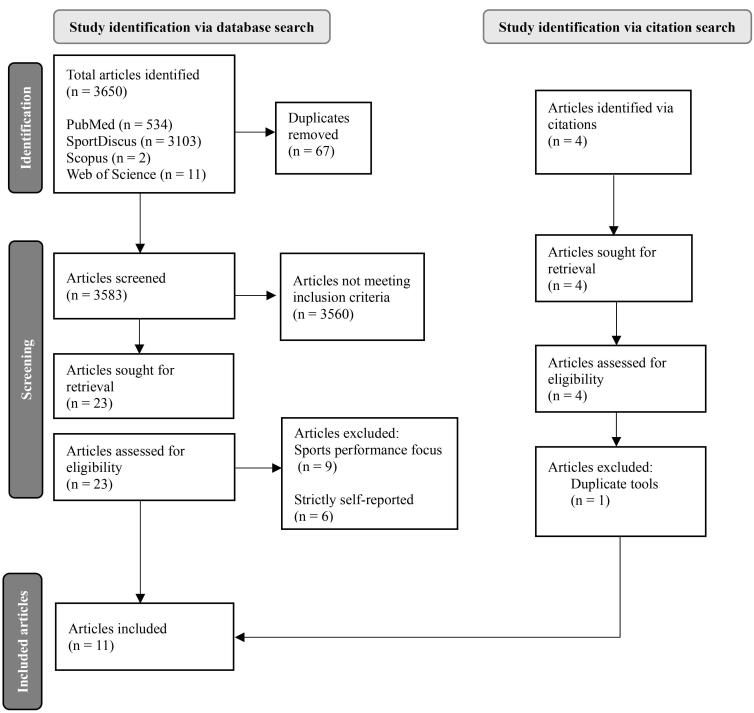
Rapid review flow chart

 The search yielded 3650 articles (PubMed *n*= 534; SportDiscus *n*= 3101; Scopus *n*= 2; Web of Science *n*= 11). After removing duplicates, 3583 titles were screened with 23 articles retrieved for full assessment. Eleven relevant articles were retained, 8 covering tool development and 3 describing validation and field-testing, see Figure 1 for the PRISM flow chart of included articles.

 Inductive thematic analysis generated four tool categories: 1) coaching behaviour and motivational climate; 2) referee-athlete interaction; 3) injury surveillance and 4) alcohol management. Three observational tools were designed to measure coaching behaviour and motivational climate in sports settings: the Coach Analysis and Intervention System (CAIS),^45^ the Assessment of Coach Tone (ACT)^46^ and the Multidimensional Motivational Climate Observation System (MMCOS).^47^ These tools were developed to observe and analyse coaching behaviours to promote sport participant health. Their development began with literature reviews to establish content, followed by expert and field actor input and feedback on final versions and then pilot tested and validated by sports coaches.^48,49^ The Referee-Players’ Interaction Assessment Scoring System tool (R-PIASS)^50^ was the only included study that developed a measurement tool to assess referee-athlete interactions and includes an action framework. This tool’s development began with reviewing both existing literature and empirical studies. The R-PIASS includes 6-dimentions of referee-athlete interactions, requiring observers to undergo extensive training to record and code observed interactions. It was developed and validated for use in handball^50^ and field tested in rugby.^51^ The injury surveillance category included three tools: the Drop Vertical Jump Screening test^52^ assessing ACL injury risk, the Youth Sports Injury Survey tool^53^ which captures athlete injury exposure and the Ice Hockey Injury Surveillance tool^54^ that monitors youth hockey injuries. Their development was based on field observations^52^ and adaptations from pre-existing surveillance tools.^53,54^ While the Drop Vertical Jump Screen test was directed towards physicians and allied health professionals, necessitating training in physiology,^52^ the other two are usable by sports club actors. The alcohol management category included one tool: the Alcohol Management Observational Audit tool, a 13-item checklist that audits alcohol management practices in community football clubs.^19^ It was developed as part of the ‘Good Sport’ programme in Australia to encourage grassroots sports clubs to provide healthy and safe environments in all clubs and during events.^55^ For details, see Table S1 Rapid Review Study Details.

 Although useful, these tools do not directly observe the HPSC as a setting. They primarily focus on observations of sports actor behaviours at the coaching level^45,46,50^ or on specific health topics such as injury,^52-54^ motivation^47^ or alcohol management.^19^ Additionally, the four HPSC health determinants^17^ were not considered. Furthermore, some tools required extensive training^46,47,52^ and reporting for proper observation^53,54^ rendering them complicated for sports club actors. Therefore, an HP observational tool, grounded in theory, is essential for sports managers to directly assess HP in their club.

###  Step 2: Delphi Study Results

####  Results Round 1

 Round 1 included 18 experts from the HEPA Europe network working group (see Table 1 for respondent characteristics). Checklist consensus was reached with 24 items separated into 4 observation areas: ‘Locker rooms’ (*n*= 5), ‘Website’ (*n*= 12), ‘Cafeteria & Food Offerings’ (*n*= 2) and ‘Facilities & Surroundings’ (*n*= 5). The ‘Cafeteria & Food Offerings’ area items were adapted from the Alcohol Audit tool.^19^

**Table 1:** Delphi Study Respondent Characteristics by Round

**Round 1**		**Round 2**		**Round 3**	
Gender	Female *n* = 9 Male* n =*9	Gender	Female *n =*13 Male *n =*18	Gender	Female *n =*11 Male *n =*10
		Age	18 - 25 *n =*0 26 - 35 *n =*9 36 - 45 *n =*9 46 - 55 *n =*8 56 - 65 *n =*3 66 ≤ *n =*2	Age	18 - 25 *n =*0 26 - 35 *n =*4 36 - 45 *n =*8 46 - 55 *n =*6 56 - 65 *n =*2 66 ≤ *n =*1
Country	France *n =*3 Sweden *n =*4 Denmark *n =*2 Belgium *n =*2 Ireland *n =*2 United Kingdom *n =*1 The Netherlands *n =*1 Slovenia *n =*1 Italy *n =*1 Luxembourg *n =*1	Country	France *n =*15 Sweden *n =*3 Denmark *n =*2 Belgium *n =*2 Ireland *n* = 2 United Kingdom *n =*1 The Netherlands *n =*1 Slovenia *n =*4 Luxembourg *n =*1	Country	France *n =*8 Sweden *n =*2 Denmark *n =*2 Belgium *n =*2 Ireland *n* = 2 United Kingdom *n =*1 The Netherlands *n =*1 Slovenia *n =*3
Position	Academic *n =*13 Public Institute *n =*3 International/National Sports Organisation *n =*2	Position	Academic *n =*13 International/National Sports Organisation *n =*2 Member of a national or local public institution working on health promotion/sport *n* = 1 Local Sports Club Manager *n* = 15	Position	Academic *n =*11 International/National Sports Organisation *n =*2 Member of a national or local public institution working on health promotion/sport *n* = 1 Local Sports Club Manager *n* = 5 Other *n* = 2
Sport practiced /managed		Sport practiced /managed	Individual *n* = 33 Team *n* = 15 Multisport *n* = 5 No response *n* = 2	Sport practiced /managed	Individual *n* = 23 Team *n* = 9 Multisport *n* = 3 No response *n* = 2

####  Results Round 2

 Round 2 included 31 respondents representing a variety of individual (i.e., swimming, skiing) and team sports (i.e., basketball, volleyball) (see Table 1 for respondent details). Based on respondent feedback, the area ‘Locker room’ was modified to ‘Locker & Restrooms’; items that combined both terms were subsequently separated. Based on indicator ratings, one item was deleted because it fell below 80% consensus for relevance and importance, three items were reformulated and four new items were added. Most items in this area had an IQR ≤ 1, indicating strong consensus among respondents. One item, “*Personal items can be stored safely in the locker rooms*”, which had an IQR of 3 for both relevance and importance indicating low consensus among respondents, this item was below consensus thresholds and was deleted. Within the ‘Website’ area, one itemfell below consensus for relevance and importance and was deleted. Another item was reformulated for clarity, and one additional item was added: “*Based on website consultation, I can identify where the sports club’s facilities are and how to get to my sports practice*”. Respondents mentioned this area contained too many items therefore, through researcher discussions, a second item regarding the strategic vision of clubs, was deleted. All items in this area had IQRs ≤ 1 for all three indicators signifying strong consensus among respondents. In the ‘Cafeteria & Food Options’ area, one item was reformulated and nine items were added. Most additions involved providing healthy food options and responsible alcohol serving for example, “*If the sports club has at least one vending machine, it offers healthy food options*” and “*The sports club does not conduct any drinking supportive events, like happy hours or drinking competitions*”. IQRs in this area were all ≤ 1, indicating strong consensus among respondents. Four items in the ‘Facilities & Surroundings’ area were deleted. A fifth item, “*The sport club has an emergency procedure, which can be found on the premises*”, was reformulated by adding Automated External Defibrillator (AED) and first-aid examples. Based on feedback, 9 items were added covering topics such as the presence of non-smoking signage and the cleanliness/maintenance of infrastructures. This area had the largest IQR spread for three items being ≥ 2 for the feasibility indicator and one item, “*The sports clubs is used to sharing equipment or sport materials, as described in the facilities*”, having and IQR ≥ 2 for all three indicators, suggesting low consensus among participants. Several respondents commented that often grassroots sports clubs do not own their own facilities making it less feasible for them to have the ability to modify the infrastructure. Round 2 ended with 43 items: ‘Locker & Restrooms’ (*n*= 11), ‘Website’ (*n*= 11), ‘Cafeteria & Food Options’ (*n*= 11) and ‘Facilities & Surroundings’ (*n*= 10). For details, see Table S2 Delphi Study Round 2 Item Indicator Ratings and Median Scores.

 During round 2, median scores for the ‘Locker room’ and ‘Website’ areas were generally higher on all three indicators for sports managers compared to experts (researchers, academics, public health professionals). On the contrary, comparisons showed that experts tended to score items in the ‘Cafeteria & Food Options’ and ‘Facilities & Surroundings’ areas approximately 1-point higher on indicators compared to sports managers. See Table S3 Group Median Score Comparisons for details.

####  Results Round 3

 Round 3 included a total of 21 respondents representing a variety of individual and team sports (see Table 1 for respondent characteristics). Four items from the ‘Locker & Restrooms’ area were deleted falling below consensus for all three indicators, two items were reformulated and two items were added. Researchers discussed an additional two items that reached 79% consensus for feasibility, they were retained due to high scores for the other two indicators. Furthermore, the term* ‘*discrimination’ was understood differently by respondents, some interpreting it as race or sexual orientation and others about disabilities. Therefore, items were added to make the distinction in both locker rooms and restrooms. IQRs in this area had the largest spread with six items ≥ 2 for the feasibility indicator, three items ≥ 2 for relevance and five items ≥ 2 for importance indicating low respondent consensus. One item, “*People in the locker room greet you when entering the locker rooms*”, was ≥ 3 on all indicators. The ‘Website’ area had two items deleted, while two additional items were discussed for achieving 79% consensus for feasibility but retained due to higher scores for the other two indicators. This area had the lowest IQR spread with two items ≥ 2 on one indicator and one item, “*The website includes a code of conduct or health promotion charter for sports participants, parents or spectators”,* ≥ 2 on all three indicators suggesting low respondent consensus. The ‘Cafeteria & Food Options’ area had two items deleted, one item reformulated and five items were discussed for low feasibility scores but high importance and HP relevance scores. For example, “*The display of items in the cafeteria are in favour of healthy options*”, received 79% for feasibility but 95% for the other two indicators. Nine items in the area had IQRs of ≥ 2 on the feasibility indicator with three of these items ≥ 2 for all three indicators. Respondents mentioned that many sports clubs do not have a cafeteria or that they do not have control over the food that is offered, making these items less relevant. Finally, four items in the ‘Facilities & Surroundings’ area were deleted, one item was reformulated, another was separated into two and two additional items were added based on respondent feedback. For example, “*The sports club has a first aid kit or heart defibrillator visible on the sports club premises*” received high scores but respondents suggested separating it as some sports clubs may have a first aid kit or an AED but not both. This area had two items with IQRs ≥ 2 on all three indicators and a third item, “*The sports clubs proposes different options to reduce carbon emissions, like car sharing or public transportation information*” with IQR ≥ 2 only on the importance indicator. Again, respondents commented that many sports clubs do not have the ability to modify their structure so these items may not be relevant, feasible or important for them. All three of these items were deleted due to low respondent consensus. At the end of round 3, the checklist included 36 items: ‘Locker & Restrooms’ (*n*= 9), ‘Website’ (*n*= 9), ‘Cafeteria & Food Options’ (*n*= 9) and ‘Facilities & Surroundings’ (*n*= 9). For details see Table S4 Delphi Study Round 3 Item Indicator Ratings and Median Scores.

 Round 3 median score comparisons between the expert group and sports managers were similar with no area displaying significant overall differences. Most items in the ‘Locker & Restrooms’ and ‘Website’ areas had slightly lower median scores (0.5 difference) from experts compared to sports managers. Only one item, “*On the website, I can identify my coach and how he promotes health*”, had a median score difference with sports managers rating it 1.5 higher on all three indicators. On average, median score comparisons in the ‘Cafeteria & Food Options’ and ‘Facilities & Surroundings’ areas were almost equal for all indicators with expert median scores being slightly higher by 0.5. Only two items, “*The sports club provides healthy food options in the cafeteria*” and “*The sports club facilities are available outside of training or competition for everyone*” had notable differences with expert median scores being significantly higher than sports managers. For details, see Table S3 Group Median Score Comparisons.

###  Step 3: Item Categorization

 Five HPSC researchers contributed to item categorization and seven for consensus. In the ‘Website’ area, three HPSC health determinants were targeted: Economic (*n*= 2), Organizational (*n*= 7) and Social (*n*= 6). In the ‘Facilities & Surroundings’ area, all four health determinants were targeted: Economic (*n*= 1), Environmental (*n*= 6), Organizational (*n*= 4) and Social (*n*= 1). In the ‘Locker & Restrooms’, three health determinants were targeted: Environmental (*n*= 7), Organizational (*n*= 1) and Social (*n*= 2). Finally, in the ‘Cafeteria & Food Options’ area, three health determinants were targeted: Economic (*n*= 2), Environmental (*n*= 3) and Organizational (*n*= 5). For details, see Table S5 Items Categorized into HPSC Health Determinants.

 Once categorization was complete, a finalization meeting was organised with 22 HEPA experts to validate the structure, layout and final content of the checklist. The order of the areas was modified beginning with ‘Website’ followed by the other areas. Then, experts drafted a user’s guide with an evaluation system designed for sports managers to visit each area and check if items are visibly ‘present’. Once all areas are observed, scores are calculated with ‘Low’ (*n*< 4), ‘Medium’ (*n*= 4-6) or ‘High’ (*n*> 6) based on the cumulative number of ‘Present’ checks per area. Areas receiving ‘Low’ evaluations are deemed high priority with evidence-based recommendations provided. The guide recommends the tool be used at least twice per sporting season recording evaluation dates, changes and goals for improvement. Results can be compared across one season or multiple seasons to increase the health and well-being of sports club actors and the surrounding community.

## Discussion

 To embrace the settings-based approach to HP, sports managers should observe their club using a systems-based approach by incorporating HP throughout policies, objectives and daily activities. This aligns with previous research regarding responsible alcohol management^19,55^ and rejecting junk food sponsorships^56^ in favor of healthy food options.^9,57^ HP should be systematically embedded throughout the sports club,^16^ so that HP is implicitly incorporated in all actions and attitudes and explicitly demonstrated by actors and stated within club policies.

 Combined, the rapid literature review and 3-round Delphi study achieved European consensus from 8 countries to develop and validate content of a practical HPSC Observational tool measuring key components of HP in sports clubs. This is the first observational tool grounded in the HPSC approach,^17^ covering the four HPSC health determinants specifically directed towards grassroots sports clubs. The tool provides sports club actors with a practical method of observing and improving HP found to be crucial in previous research.^58,59^

 Rapid review results found that all 8 observational tools began development with literature reviews, called upon expert input and then tested in target populations. Many of the tools require extensive training,^45-47,52^ are focused on one health topic^19,52-54^ or are directed at specific populations^49,50,54^ or sports.^50,54^ Results from the current rapid review and previous literature reviews highlight that sports clubs focus on performance and injury prevention^58,60,61^ and thus have difficulties understanding and implementing a broader view of HP in the sports context.^18^ Similar to the development process found in the rapid review, the HPSC Observational tool began with reviewing the literature, followed by input from sports managers and experts. In contrast, the developed tool takes a broad view of observable HP elements within sports clubs rather than taking a biomedical approach^52^ or focusing on specific behaviours.^45-47,50^ Additionally, it has a binary scoring system rendering it easy to use with minimal bias whereas some of the rapid review tools require observers with knowledge in physiology such as the Drop Vertical Jump Screening Test^52^ or extensive training to code behaviours as in the CAIS and ACT tools.^45,46^ The only tool found to assess the HP environment was the Alcohol Management Observational Audit tool.^19^ Although useful, this tool assesses only one HP topic, alcohol provision, rather than observing multiple HP topics.

 Within the HPSC model, the environmental health determinant describes the built surroundings of sports clubs with the social determinant describing the norms and culture of the club.^39^ Other definitions include a more holistic vision incorporating the social environment created through dynamic interpersonal relationships and cultural milieus.^62^ The physical environment often depends on cultural and social factors formed through human behaviours and dynamic relationships.^63,64^ Mechanisms by which the built environment affects individual behaviours have been studied with several hypothesis suggesting mental health and well-being, physical activity levels and social connections can be altered depending upon physical spaces.^65,66^ For example, the Senior Walking Environmental Assessment tool and the Residential Environment Assessment tool, were developed to determine effects of the built environment on physical, social and mental health and well-being.^67,68^ These examples display how the built environment not only promotes physical health but is interwoven with social factors to promote broader health determinants.

 Even though the HPSC Observational tool is based on the HPSC model, social and organisational constructs are only implicitly incorporated, as items were crafted based on location rather than the theoretical model. Although social determinants of health include components of the built environment, they also incorporate social and community contexts.^69^ Previous studies researching how built environments elicit physical activity noted that social and cultural constructs around physical activity participation were as important as providing built structures for participation.^70,71^ This questions whether focusing solely on explicit HP elements in the environment is sufficient to define a setting as health-promoting, highlighting the need to include implicit aspects of HP and an understanding that settings-based HP is a process inclusive of multiple health determinants interacting within closed and open systems.^12^ Strict observational tools capture a snapshot of one point in time but less is known about capturing the many nuances of a comprehensive health-promoting setting. While the HPSC Observational tool can be used alone, it is also part of a broader resource toolkit for HP assessment, training and education within the sports context to advance the HPSC approach. For example, the e-PROSCeSS questionnaire assesses perceptions of HP from the point-of-view of managers, coaches and sports participants,^22^ while the PROSCeSS MOOC was developed to educate sports club actors about the importance of HP within their sports club.^72^ Additional tools offer sports coaches,^30^ federations^73^ and managers^74^ guidance into the complex process of becoming a comprehensive health-promoting sport club by incorporating health into all of their activities, actions and decisions.^12^

## Limitations

 Some study limitations must be acknowledged. Although a rapid literature review was conducted to search for HP observational tools in sports settings, other settings could have yielded useful tools. To minimize this limitation, the search included all sports contexts rather than limiting it to sports clubs. A second limitation concerns the respondents that chose to participate in the study. Participants were invited through a network of experts working on HPSC, which could limit views based on selection bias. Researchers attempted to minimize this limitation by soliciting sports manager participation. A third limitation involves the attrition of respondents between round 2 and round 3 of the Delphi study. Round 3 saw a 38% attrition rate from the previous round which could have impacted the final item list. Efforts were taken to retain as many respondents as possible including sending reminder emails with the survey link. Additionally, researchers discussed the final list with the HEPA experts to ensure content validation. Last, the classification of items into health determinants was limited to HPSC researchers; results may have varied if this was conducted during the Delphi study. A deliberate choice was made to limit the Delphi study scope for reasons of health determinant comprehension by field actors, time constraints and respondent fatigue.

## Conclusion

 For over a decade, researchers and government agencies have requested expanding the settings-based approach to HP through developing innovative settings such as sports clubs.^18,34,38^ Observational tools are powerful means to directly assess HP in real-world contexts. This study provides a tool aligned with goals from the HPSC approach to improve the health-promoting environment within sports clubs. Sports managers can observe HP changes within specific areas of their club over time aiming for small but targeted improvements. Psychometric validation is in process through a test/re-test method in multiple European sports clubs. Psychometric validation will include a survey regarding usefulness, feasibility and sustainability of the observational tool. Once validated, this HPSC Observation Tool will be disseminated through project partner networks including sports federations, university courses, local sports club associations and the HEPA Europe network. Widespread dissemination will directly help sports clubs to improve the current state of HP within their club and could be used to develop HP policies and actions.

## Competing Interests

 The following manuscript was conducted as independent research and analysis. Authors have no conflicts of interest to declare.

## Ethical Approval

 The study was approved and registered with the National Commission of Informatics and Freedom (CNIL), by the data protection officer from the Université de Lorraine, under N°2025-412. All participants freely agreed to participate and signed an informed consent prior to participation in each round of data collection. The review protocol was registered with PROSPERO: CRD42024621443.

## Supplementary File

Supplementary file contains Tables S1-S5.
