# Supplementary material for: Development and Content Validation of The Health Promoting Sports Club Observational Tool
Source: Health Promot Perspect. 2026 Jun 6;16(1):63–73. doi: 10.34172/hpp.45562 (PMC13402225; doi:10.34172/hpp.45562)
Supplement: Supplementary file 1 — Supplementary file contains Tables S1-S5. [file hpp-16-63-s001.pdf]

Johnson et al, Health Promotion Perspectives 2026;16(1):63-S1
doi:10.34172/hpp.45562
https://hpp.tbzmed.ac.ir

Table S1. Rapid Review Study Details

Authors	Article title	Country	Tool name	Acronym	*Quality rating/Risk of bias	Theme	HPSC targeted level	Health Determinant targeted	Development design	Content/Structure	Target population	Environment deployed	Validation method
Brust et al., 1992[54]	Children's Ice Hockey Injuries	United States	Ice hockey Injury Surveillance Tool (unofficial name)	None	3.64/Low	Injury surveillance	Individual, Micro, Meso	Environmental, Organisational	Adapted from Gerberich's injury reporting scale with added "minimal injury" category; data collected by coaches, managers and trained observers	Detailed data collection regarding injury event (date, location, player info), context (position, skill level), injury type, mechanism (e.g., contact), and penalties. Used alongside surveys and follow-up with parents for injury confirmation and clarification	Youth ice hockey actors	Youth match settings	Three phase validation: (1) Multi-observer documentation of injuries; (2) Surveys from coaches, players and parents on injury awareness; (3) Post-injury verification by a research assistant, medical release and parental confirmation
Cushion et al., 2012[45]	Developing the Coach Analysis and Intervention System (CAIS): Establishing validity and reliability of a computerised systematic observation instrument	United Kingdom	Coach Analysis and Intervention System	CAIS	3.60/Low	Coaching behaviour	Meso	Social	Sports Code analysis software; literature review, expert feedback, pilot testing and coder training	6-step coding of 23 primary coaching behaviors categorized into 8 themes	Youth coaches	Youth practices and competitions	Validity and reliability established via expert feedback, content validity through pilot and amendments, intra- and inter-observer reliability using movie-testing, expert face validity

Erickson and Côté, 2015[46]	The Intervention Tone of Coaches' Behaviour: Development of the Assessment of Coaching Tone (ACT) Observational Coding System	United States	Assessment of Coach Tone	ACT	3.88/Low	Coaching behaviour	Micro	Social	Six-stage development and validation: expert input, iterative refinement, and coder training using Noldus software; pilot tested to establish generalizability	Behaviour coding includes one content code and up to two tone modifiers; structured sequences categorize tone-related coaching behaviors; adaptable to live or computerized coding format	Youth coaches	Competitive youth sports settings	Three phase validation: (1) Multi-observer documentation of injuries; (2) Surveys from coaches, players and parents on injury awareness; (3) Post-injury verification by a research assistant, medical release and parental confirmation
Erickson and Côté, 2015[48]	A season-long examination of the intervention tone of coach–athlete interactions B5	United States	Assessment of Coach Tone	ACT	3.86/Low	Coaching behaviour	Micro	Social	Six-stage development and validation: expert input, iterative refinement, and coder training using Noldus software; pilot tested to establish generalizability	Behaviour coding includes one content code and up to two tone modifiers; structured sequences categorize tone-related coaching behaviors; adaptable to live or computerized coding format	Youth coaches	Competitive youth sports settings	Testing the previously validated tool over one sporting season
McFayden et al., 2019[19]	Sustaining the implementation of alcohol management practices by community sports clubs: a randomised control trial	Australia	Alcohol management observational audit tool	None	3.50/Low	Alcohol management	Individual, Macro	Economic, Environmental, Organisational, Social	Developed as part of a web-based intervention in the Good Sports programme. Piloted in 4 clubs. Based on methods from 200+	13-item observational checklist assessing alcohol management (e.g., responsible service, signage, intoxication management)	Non-elite community football clubs	Senior football games at local clubs	Two trained research assistants audited clubs twice. Discrepancies resolved via discussions using objective criteria (e.g., signage, signs

									prior observations				of intoxication). Standardized one-day training included scenario-based exercises
Płoszaj, et al., 2020[50]	The Referee as an Educator: Assessment of the Quality of Referee-Players Interactions in Competitive Youth Handball	Poland	Referee-Players' Interaction Assessment Scoring System tool	R-PIASS	3.56/Low	Referee-athlete interaction	Individual, Micro	Social	Literature review; empirical studies; structured observations; test piloted	6-dimensions: Positive Climate, Responsiveness, Behaviour Management, Proficiency, Instructing, and Communicating. Observers record interactions pre-, during-, and post-match periods using a scoring sheet. Each dimension is rated on a 7-point scale	Handball referees	Competitive youth handball	Internal consistency (Cronbach's alpha), inter-rater reliability (≥80% agreement), exploratory factor analysis; subsequent study in youth rugby to test applicability and sensitivity across sports
Płoszaj, et al., 2021[51]	Positive Behavior Management: Assessment of Rugby Referees in Children Sport	Poland	Referee-Players' Interaction Assessment Scoring System tool	R-PIASS	3.62/Low	Referee-athlete interaction	Individual, Micro	Social	Literature review; empirical studies; structured observations; test piloted	2-dimentions used in this study: positive behaviour management; proficiency	Rugby referees	Children's rugby	Testing the previously validated tool in a different sport for generalizability
Radelet, et al., 2002[53]	Survey of the Injury Rate for Children in Community Sports	United States	Youth Sports Injury Survey Tool (unofficial name)	None	2.64/Moderate	Injury surveillance	Individual, Micro, Meso, Public health actors	Environmental, Organisational	Developed using pilot data from youth baseball and soccer teams; refined through coach feedback and pilot tested across multiple	Survey tool uses checklists and team rosters. Coaches report injuries, participation numbers, injury location, cause, type, care received and game/practice context. Roster includes age, gender, player initials, and exposure time. Designed to be simple and coach-administered with ongoing	Youth coaches	Youth practice and game settings	Validated through expanded multi-sport implementation. Coaches submitted weekly data; researchers calculated injury rate per 100 athlete exposures,

									sports. Coaches were trained and monitored for weekly reporting compliance	researcher oversight for compliance			conducted logistic regression analysis, and used FITS (frequency of injury per team per season) to examine injury risk. Reliability ensured via coach training and consistent researcher follow-up
Redler, et al., 2015[52]	Reliability of a Field-Based Drop Vertical Jump Screening Test for ACL Injury Risk Assessment	United States	Drop Vertical Jump Screening Test	None	3.95/Low	Injury surveillance	Individual, Micro, Public health actors	Environmental, Organisational	Developed using recorded jump trials of 15 youth athletes and a panel of healthcare professionals	Participants jump from a 12-inch box onto a marked floor, then perform a vertical jump while being recorded. Observers evaluate video for dynamic knee valgus and assign risk categories (low/medium/high) based on movement patterns	Physicians and allied health professionals including sports coaches	Controlled field-based setting on youth athletes between 11-17 years old	Observer ratings of injury risk were compared with objective knee valgus data (Dartfish software) to assess sensitivity and specificity. Intra-observer reliability was tested with a 6-week follow-up
Smith et al., 2015[47]	Development and Validation of the Multidimensional Motivational Climate Observation System	International	Multidimensional motivational climate observation system	MMCOS	3.90/Low	Motivational climate Coaching behaviour	Micro	Social	Three-stage development and validation: expert review, statistical validation of constructs, and inter-coder reliability testing in	Hierarchical system with 2 higher-order dimensions (empowering/disempowering), 7 environmental dimensions, and 32 specific strategies; uses 4-point potency scale + checklist for behavioural indicators	Youth coaches	Training and competition contexts in grass-roots youth sport	Content validity confirmed by expert review; construct validity verified by pilot and statistical analyses; reliability assessed via inter-coder

									training and competition				agreement in training and competition
Smith et al., 2017[49]	Observing the coach-created motivational environment across training and competition in youth sport	International	Multidimensional motivational climate observation system	MMCOS	3.12/Moderate	Motivational climate Coaching behaviour	Micro	Social	Pilot test of the MMCOS	Coding coach behaviors as empowering or disempowering	Youth coaches	Training and competition of youth soccer	Field testing

*Quality rating based on a 4-point rating scale with 1 = low, 2 = fair, 3 = good and 4 = high / Risk of bias = Low, Moderate, High

Table S2. Delphi Study Round 2 Item Indicator Ratings and Median Scores

**Round 2**	**% Relevance**	**% Feasibility**	**% Importance**	**Median (IQR) Relevance**	**Median (IQR) Feasibility**	**Median (IQR) Importance**
**Locker room**						
‡The locker room and restroom is clean	87	97	90	6 (1)	5.5 (1)	5 (1)
‡The locker room and restroom is accessible to every member of the sports club without discrimination	93	97	93	6 (1)	5 (1)	6 (1)
*Personal items can be stored safely in the locker rooms	67	83	63	5 (3)	5 (0)	5 (3)

‡There are posters or displays on how to make the locker room clean, safe and accessible to all	83	90	83	5 (1)	5 (1)	5 (1)
There is opportunity to take a safe and private hot shower in the locker room	90	93	87	5 (1)	5 (1)	5 (1)
**Website**						
The website contains a webpage or a section on health (social, physical, mental)	93	97	93	5 (1)	5 (1)	5 (1)
*The website contains a strategic plan or strategic vision of how the sports association should promote health	90	90	93	5 (1)	5 (1)	6 (1)
The website includes a code of conduct or health promotion charter for sports participants, parents or spectators	100	100	100	5 (1)	5 (1)	5 (1)
The website provides contact details of a person responsible for	90	87	90	6 (1)	6 (1)	6 (1)

health at the sports club						
‡The website identifies the committee or group responsible for health in the sports club	93	87	93	5 (1)	5 (1)	5 (1)
Based on website consultation, I can identify participation opportunities for non-competitive sport practice	87	100	87	5 (1)	5 (1)	5 (1)
*Based on website consultation, I can identify flexible, one-time sport participation opportunities	77	83	77	5 (1)	5 (1)	5 (1)
‡Based on website consultation, I can identify specific rates for deprived participants	87	90	90	5 (1)	5 (1)	5 (1)
Based on website consultation, I can identify mention of events or activities related to promoting health in the sports club	100	100	100	6 (1)	6 (1)	5 (1)

Based on website consultation, the sport clubs mentions collaborations with local charities, health or social organisations or health professionals	97	90	93	5 (1)	5 (1)	5 (1)
The website contains information, resources or guidance on at least one health promotion topic	97	87	93	5 (1)	5 (1)	5 (1)
On the website, as a member of the sport club, I can share my voice or report information by contacting a sport club manager	90	87	90	5 (1)	5 (1)	5 (1)
**Facilities & Surroundings**						
*In the sports club, sponsorship does not include unhealthy services or offers from the provider	73	63	73	5 (1.5)	5 (2)	5 (1.5)
*Sports club facilities are located in a walking and	90	60	80	5 (1)	5 (3.25)	5 (1)

cycling friendly environment						
‡The sport club has an emergency procedure, which can be found on the premises	83	87	83	6 (1)	6 (1)	6 (1)
*The sports club has recycling options for waste management	83	77	83	5 (1)	5 (1)	5 (1)
*The sports clubs is used to sharing equipment or sport materials, as described in the facilities	57	60	60	5 (2)	5 (2)	5 (2.25)
**Cafeteria & Food Options**						
§Free tap water is available at the sports club	90	90	90	6 (1)	6 (0.25)	6 (1)
The sports club has a policy or charter on responsible alcohol serving	87	87	87	5 (1)	5 (1)	5 (1)

*items deleted; ‡items reformulated; §items added

Table S3. Group Median Score Comparisons

**Round 2**	**Expert median scores**			**Sports Manager median scores**		
**Locker & Restrooms**	Relevance	Feasibility	Importance	Relevance	Feasibility	Importance
Item 1: The locker room and restroom are clean	5	5	5	6	6	5.5
Item 2: The locker room and restroom are accessible to all members of the sports club without discrimination	5	5	5	6	6	6
Item 3: Personal items can be store safely in the restrooms	3	5	3	5	5	5
Item 4: There are posters or displays on how to make the restroom and locker room friendly. safe and accessible for all	5	5	5	5	5	5
Item 5: There is an opportunity to take a hot shower in the locker room	5	5	5	6	6	6
**Website**	Relevance	Feasibility	Importance	Relevance	Feasibility	Importance
Item 1: The website contains a webpage or a section on health (social. physical. mental)	5	5	5	6	6	6
Item 2: The website contains a strategic plan or strategic vision of how the sports association should promote health	6	5	6	5	5	5
Item 3: The website includes a code of conduct or health promotion charter for sports participants. parents or spectators	6	5.5	6	5	5	5
Item 4: The website provides contact details of a person responsible for health at the sports club	5.5	5	5	6	6	6
Item 5: The website identifies the committee or group responsible for health in the sports club	5	5	5	5	5	5
Item 6: Based on website consultation, I can identify participation opportunities for non-competitive sports practice	5	5	5	6	6	6
Item 7: Based on website consultation, I can identify flexible, one-time sport participation opportunities	3	5	4	6	6	6
Item 8: Based on website consultation, I can identify specific rates for deprived participants	5	5	5	5	5	5

Item 9: Based on website consultation, I can identify mentions of events or activities related to promoting health in the sports club	5.5	5	5	6	6	6
Item 10: Based on website consultation, the sport clubs mentions collaborations with local charities, health or social organisations or health professionals	5	5	5	6	6	6
Item 11: The website contains information, resources or guidance on at least one health promotion topic	5	5	5	5	5	5
Item 12: On the website. as a member of the sport club, I can share my voice or report information by contacting a sport club manager	5.5	5	6	5	5	5
**Cafeteria & Food Options**	Relevance	Feasibility	Importance	Relevance	Feasibility	Importance
Item 1: Free tap water is available at the sports club	6	6	5.5	6	6	6
Item 2: The sports club has a policy or charter on responsible alcohol serving	6	6	6	5	5	5
**Facilities & Surroundings**	Relevance	Feasibility	Importance	Relevance	Feasibility	Importance
Item 1: In the sports club, sponsorship does not include unhealthy services or offers from the provider	5	5	5	5	5	5
Item 2: Sports club facilities are located in a walking and cycling friendly environment	5	4	5	5	5	5
Item 3: The sport club has an emergency procedure, which can be found on the premises	6	6	5.5	5.5	6	6
Item 4: The sports club has recycling options for waste management	5	5	5	5	5	5
Item 5: The sports clubs is used to sharing equipment or sport material as described in the facilities	5	5	5	4	4	4
**Round 3**						
	**Expert median scores**			**Sports Manager median scores**		
**Locker & Restrooms**	Relevance	Feasibility	Importance	Relevance	Feasibility	Importance
Item 1: The locker rooms are clean and well-maintained	5	5	5	5	5	5
Item 2: The rest rooms are clean and well-maintained	5	5	5	5	5	5
Item 3: The locker rooms are accessible to all members of the sports club without discrimination	5	5	5	5	5	5

Item 4: The rest rooms are accessible to all members of the sports club without discrimination	6	4	5	5	5	5
Item 5: There are posters or displays on how to make the rest rooms or locker rooms clean. safe and accessible for all	5	5	5	4	3	4
Item 6: There is an opportunity to take a hot shower in the locker room	5	5	5	6	5	6
Item 7: Basic hygiene items (e.g. soap. toilet paper) are available in the locker or restrooms	5	5	5	5	2	5
Item 8: There are sanitary protections (e.g. sanitary napkins. tampons) available in the locker or restrooms	5	4	5	6	2	6
Item 9: There is an opportunity to take a safe and private shower in the locker rooms	5	4	5	5	5	5
Item 10: There is a sign posted restricting on the use of smartphones. cameras or laptops in the locker or restrooms	5	5	5	3	2	3
Item 11: People in the locker room greet you when entering the locker rooms	3	3	3	6	5	5
**Website**	Relevance	Feasibility	Importance	Relevance	Feasibility	Importance
Item 1: The website contains a webpage or a section on health (social. physical. mental) and/or the benefits of sport practice	5	5	5	4.5	4.5	5
Item 2: The website includes a code of conduct or health promotion charter for sports participants, parents or spectators	5	5	5	4.5	4.5	4.5
Item 3: As a member of the sports club. I can share my voice or report information by contacting sports club management through the website	5	5	5	5	5	5
Item 4: The website provides information and contact details of a person or committee responsible for health in the sports club	5	5	5	4.5	4.5	5
Item 5: Based on website consultation, I can identify participation opportunities for non-competitive sport practice and see that all skill levels are welcome	5	5	5	4.5	4.5	5
Item 6: Based on website consultation, I can identify where the sports club's facilities are and how to get to my sports practice	5	5	5	5	5	5

Item 7: Based on website consultation, I can identify specific membership rates for underprivileged participants (people with low socio-economic background, people with disabilities...)	5	5	5	5	5	5
Item 8: Based on website consultation, I can identify mentions of events or activities related to promoting health in the sports club	5	5	5	5	5	5
Item 6: Based on website consultation, I can identify where the sports club's facilities are and how to get to my sports practice	5	4	5	5	5	5
Item 10: The website contains information, resources or guidance on at least one health topic	5	5	5	5	5	5
Item 11: On the website, I can identify my coach and how he promotes health	3	3	3	4.5	4.5	4.5

**Cafeteria & Food Options**	Relevance	Feasibility	Importance	Relevance	Feasibility	Importance
Item 1: There is a drinking fountain or free tap water available at the sports club	6	5	6	5	4	5
Item 2: The sports club has a code of conduct or charter on responsible alcohol serving	5	5	5	5	5.5	5.5
Item 3: If the sports club has at least one vending machine, it offers healthy food option	5	5	5	5	4	5.5
Item 4: The sports club provides healthy food options in the cafeteria	6	5	6	5	2.5	4.5
Item 5: Healthy food options have reasonable prices in comparison to unhealthy food options	6	5	6	5.5	4	5.5
Item 6: The display of items in the cafeteria are in favor of healthy options	5	4	5	5	4	5.5
Item 7: The person serving in the cafeteria greets me upon arrival	4	3	4	4	4	4
Item 8: The cafeteria is clean, well maintained, and food is stored appropriately (e.g. fridge)	5	5	5	4.5	4.5	5
Item 9: The person serving in the cafeteria is not drinking alcohol while serving	6	6	6	4.5	4	5
Item 10: People who are drunk or under the influence of drugs are not served. nor allowed to enter the sports club premises or cafeteria	6	6	6	6	5	6

Item 11: The sports club does not conduct any drinking supportive events, like happy hours, drinking competitions, etc.	5	5	5	5.5	4	5.5
**Facilities & Surroundings**	Relevance	Feasibility	Importance	Relevance	Feasibility	Importance
Item 1: In the sports club. people greet me upon arrival	5	5	5	5	5	6
Item 2: Sports club facilities include a secure bicycle or scooter parking	5	5	5	4	3	5
Item 3: The sport club has an emergency procedure (emergency number, evacuation plan...), which can be found on the premises	5	6	5	5	5	5
Item 4: The sports club has a first aid kit or a heart defibrillator visible on the sport club premises	5	5	5	5	5	5
Item 5: The sports clubs has sufficient and well maintained equipment for all of its members	5	5	5	5	5	5
Item 6: The sports club has no-smoking signage in the facilities	6	6	6	6	6	6
Item 7: The sports club infrastructures are clean and well-maintained	6	6	6	5	5	5
Item 8: The sports club facilities are available outside of training or competition for everyone	5	4	5	2	2	2
Item 9: The sports club proposes different options to reduce carbon emissions, like car sharing or public transportation information	5	4	4	5	4	5
Item 10: The spectators are encouraged to stay together in a dedicated space for competitions	4	3	4	5	5	5

Table S4. Delphi study round 3 item indicator ratings and median scores

Round 3	% Relevance	% Feasibility	% Importance	Median (IQR) Relevance	Median (IQR) Feasibility	Median (IQR) Importance
**Locker & Restrooms**						
The locker rooms are clean and well-maintained	95	89	89	5 (1.5)	5 (1.5)	5 (1.5)
The restrooms are clean and well-maintained	95	89	89	5 (1)	5 (1.5)	5 (1)
The locker rooms are accessible to all members of the sports club without discrimination	84	89	89	5 (1.5)	5 (1.5)	5 (1)
§The locker rooms are accessible to all members of the sports club including those with disabilities						
‡The restrooms are accessible to all members of the sports club without discrimination	84	74	84	5 (1)	5 (2)	5 (1)
§The restrooms are accessible to all members of the sports club including those with disabilities						
*There are posters or displays on how to make the restrooms or locker rooms clean, safe and accessible for all	63	68	63	5 (3)	5 (2.75)	5 (3)

There is an opportunity to take a hot shower in the locker room	95	84	84	5 (1.5)	5 (1)	5 (2)
Basic hygiene items (e.g. soap, toilet paper) are available in the locker or restrooms	89	79	89	5 (1.5)	5 (2)	5 (2)
*There are sanitary protections (e.g. sanitary napkins, tampons) available in the locker or restrooms	79	68	79	5 (1)	4 (2)	5 (1)
There is an opportunity to take a safe and private shower in the locker rooms	95	79	84	5 (1)	4 (1)	5 (1)
*There is a sign posted restricting on the use of smartphones, cameras or laptops in the restrooms or locker rooms	63	68	63	5 (2.5)	4 (2)	5 (2.5)
*People in the locker room greet you when entering the locker rooms	53	53	58	4 (3)	4 (3)	4 (3)
**Website**						
The website contains a webpage or a section on health (social, physical, mental) and/or the benefits of sport practice	95	89	95	5 (1.5)	5 (1)	5 (2)
*The website includes a code of conduct or health promotion charter for	84	79	84	5 (2)	5 (2)	5 (2)

sports participants, parents or spectators						
As a member of the sports club, I can share my voice or report information by contacting sports club management through the website	95	100	100	5 (1)	5 (0)	5 (0.5)
The website provides information and contact details of a person or committee responsible for health in the sports club	95	89	89	5 (1)	5 (0.5)	5 (1)
Based on website consultation, I can identify participation opportunities for non-competitive sport practice and see that all skill levels are welcome	89	84	84	5 (1)	5 (1)	5 (1)
*Based on website consultation, I can identify where the sports club's facilities are and how to get to my sports practice	79	95	79	5 (1)	5 (0)	5 (1)
Based on website consultation, I can identify specific membership rates for underprivileged participants (people with low socio-economic background, people with disabilities)	89	89	84	5 (0.5)	5 (1)	5 (1)
Based on website consultation, I can identify mention of events or	95	95	95	5 (0.5)	5 (1)	5 (1)

activities related to promoting health in the sports club						
Based on website consultation, the sports club mentions collaborations with local charities, health or social organisations or health professionals	95	79	95	5 (0.5)	5 (1)	5 (0.5)
The website contains information, resources or guidance on at least one health topic	95	95	95	5 (0.5)	5 (1)	5 (0)
*On the website, I can identify my coach and how he/she promotes health	63	53	63	4 (2)	4 (1)	4 (1.75)
**Cafeteria & Food Options**						
There is a drinking fountain or free tap water available at the sports club	95	89	89	5 (1)	5 (1.5)	5 (1)
‡The sports club has a code of conduct or charter on responsible alcohol serving	89	79	89	5 (1.5)	5 (2)	5 (2)
If the sports club has at least one vending machine, it offers healthy food options	84	68	84	5 (2)	4 (2.5)	5 (2)
The sports club provides healthy food options in the cafeteria	89	68	84	5 (1)	5 (3)	5 (1.5)

Healthy food options have reasonable prices in comparison to unhealthy food options	95	84	89	6 (1)	5 (2)	6 (1)
The display of items in the cafeteria are in favour of healthy options	95	79	95	5 (2)	4 (1)	5 (1.5)
*The person serving in the cafeteria greets me upon arrival	68	58	58	4 (2)	4 (2.5)	4 (2)
The cafeteria is clean, well maintained, and food is stored appropriately (e.g. fridge)	89	95	95	5 (1.5)	5 (2)	5 (1.5)
*The person serving in the cafeteria is not drinking alcohol while serving	79	79	74	5 (2)	5 (2.5)	6 (2)
People who are drunk or under the influence of drugs are not served, nor allowed to enter the sports club premises or cafeteria	84	79	89	6 (1)	6 (2)	6 (1)
*The sports club does not conduct any drinking supportive events, like happy hours, drinking competitions...	89	74	89	5 (1.5)	5 (2.5)	5 (2)
**Facilities & Surroundings**						
*In the sports club, people greet me upon arrival	72	72	67	5 (2.75)	5 (2.75)	5 (3)

Sports club facilities include a secure bicycle or scooter parking	94	72	89	5 (1.75)	4 (1.75)	5 (1)
The sport club has an emergency procedure (emergency number, evacuation plan...), which can be found on the premises	83	94	83	5 (1)	6 (1)	5 (1)
The sports club has a first aid kit or a heart defibrillator visible on the sport club premises	89	89	94	5 (1)	5 (1)	5.5 (1)
The sports clubs has sufficient and well maintained equipment for all of its members	89	94	94	5 (1)	5 (1.75)	5 (1.75)
The sports club has no-smoking signage in the facilities	100	100	100	6 (1)	6 (1)	6 (1)
‡The sports club infrastructures are clean and well-maintained	94	100	100	5 (1)	5 (1)	5 (1)
*The sports club facilities are available outside of training or competition for everyone	78	61	72	5 (1)	4 (1.75)	4.5 (1.75)
*The sports clubs proposes different options to reduce carbon emissions, like car sharing or public transportation information	78	67	72	5 (1)	4 (1.75)	4 (2.5)
*The spectators are encouraged to stay	56	56	67	4 (2)	4 (2)	4 (2)

together in a dedicated space for competitions				

*items deleted; ‡items reformulated; §items added for clarity

Table S5. Items Categorized into HPSC Health Determinants

	**HPSC Health Determinant Category**			
**Final items**	**Economic**	**Environmental**	**Organisational**	**Social**
**Website**				
1. The website contains a webpage or a section on health (social, physical, mental) and/or the benefits of sport practice			X	X
2. The website includes a code of conduct or health promotion charter for sports participants, parents or spectators			X	
3. As a member of the sports club, I can share my voice or report information by contacting sports club management through the website				X
4. The website provides information and contact details of a person or committee responsible for health in the sports club			X	X
5. Based on website consultation, I can identify participation opportunities for non-competitive sport practice and see that all skill levels are welcome			X	X
6. Based on website consultation, I can identify specific membership rates for underprivileged participants (e.g., people with	X			X

low socio-economic background, people with disabilities)				
7. Based on website consultation, I can identify mentions of events or activities related to promoting health in the sports club	X		X	X
8. Based on website consultation, the sports club mentions collaborations with local charities, health or social organisations or health professionals			X	
9. The website contains information, resources or guidance on at least one health topic			X	
**Facilities & Surroundings**				
1. Sports club facilities include a secure bicycle or scooter parking		X		
2. The sport club has an emergency procedure (e.g., emergency number, evacuation plan), which can be found on the premises			X	
3. The sports club has a first aid kit visible on the sports club premises		X	X	
4. The sports club has a heart defibrillator visible on the sports club premises		X		
5. The sports club has sufficient and well-maintained equipment for all its members	X		X	
6. The sports club has a “no smoking” sign in the facilities			X	X
7. The sports club facilities are clean and well-maintained		X		
8. Sports club’s facilities are accessible for people with disabilities		X		

9. The sports club has a flexible multipurpose space available with tables and chairs for meetings, seminars and educational trainings	X		
**Locker & Restrooms**			
1. The locker rooms are clean and well-maintained	X		
2. The locker rooms are accessible to all members of the sports club without discrimination	X		
3. The locker rooms are accessible to all members of the sports club including those with disabilities			X
4. The restrooms are clean and well-maintained	X	X	
5. The restrooms are accessible to all members of the sports club without discrimination	X		
6. The restrooms are accessible to all members of the sports club including those with disabilities			X
7. There is an opportunity to take a hot shower in the locker room	X		
8. There is an opportunity to take a safe and private shower in the locker rooms	X		
9. Basic hygiene items (e.g. soap, toilet paper, paper towels or hand dryer) are available in the locker or restrooms	X		
**Cafeteria & Food Options**			
1. There is a drinking fountain or free tap water available at the sports club	X		

2. The sports club’s charter on responsible alcohol serving is displayed in the cafeteria			X
3. If the sports club has at least one vending machine, it offers healthy food options	X	X	
4. The sports club provides healthy food options in the cafeteria			X
5. Healthy food options have reasonable prices in comparison to unhealthy food options	X		
6. The display of items in the cafeteria are in favour of healthy options			X
7. The cafeteria is clean, well maintained, and food is stored appropriately (e.g. refrigerator)		X	
8. People who are drunk or under the influence of drugs are not served, nor allowed to enter the sports club’s premises or cafeteria			X
9. The sports club does not conduct any drinking-supportive events, like happy hours, drinking competition, etc.			X
